# Testing the association between psychosocial job strain and adverse birth outcomes - design and methods

**DOI:** 10.1186/1471-2458-11-255

**Published:** 2011-04-21

**Authors:** Ann D Larsen, Harald Hannerz, Carsten Obel, Ane M Thulstrup, Jens P Bonde, Karin S Hougaard

**Affiliations:** 1 National Research Centre for the Working Environment, Copenhagen, Denmark; 2 Department of Occupational Medicine, Aarhus University Hospital, Aarhus, Denmark; 3 Faculty of Health Sciences, Aarhus University, Aarhus, Denmark; 4 Department of Occupational and Environmental Medicine, Copenhagen University Hospital Bispebjerg, Copenhagen, Denmark

## Abstract

**Background:**

A number of studies have examined the effects of prenatal exposure to stress on birth outcomes but few have specifically focused on psychosocial job strain. In the present protocol, we aim to examine if work characterised by high demands and low control, during pregnancy, is associated with the risk of giving birth to a child born preterm or small for gestational age.

**Methods and design:**

We will use the Danish National Birth Cohort where 100.000 children are included at baseline. In the present study 49,340 pregnancies will be included. Multinomial logistic regression will be applied to estimate odds ratios for the outcomes: preterm; full term but small for gestational age; full term but large for gestational age, as a function of job-strain (high strain, active and passive versus low strain). In the analysis we control for maternal age, Body Mass Index, parity, exercise, smoking, alcohol use, coffee consumption, type of work (manual versus non-manual), maternal serious disease and parents' heights as well as gestational age at interview.

**Discussion:**

The prospective nature of the design and the high number of participants strengthen the study. The large statistical power allows for interpretable results regardless of whether or not the hypotheses are confirmed. This is, however, not a controlled study since all kinds of 'natural' interventions takes place throughout pregnancy (e.g. work absence, medical treatment and job-redesign). The analysis will be performed from a public health perspective. From this perspective, we are not primarily interested in the effect of job strain per se but if there is residual effect of job strain after naturally occurring preventive measures have been taken.

## Background

One in eight Danish woman recently reported that she was often stressed in daily life, and workplace factors contributed significantly to the daily stress [1]. Nearly one third of all women between 25-44 years in Denmark reported in 2005 that they had difficulties completing their work tasks and 17 percent found that they only had limited or no influence on their work tasks [2].

Pregnant women, who experience distress (i.e. depression, anxiety, adverse life events, daily hassles and pregnancy-specific stress) are more likely to deliver preterm [3-6] and have a higher risk of giving birth to growth-restricted infants [6-8]. Even a moderate psychosocial load seems to increase the risk of preterm birth and stillbirth [9-12].

Several have examined the association between prenatal exposure to stress on pregnancy outcomes, but relatively few focus on the impact of the psychosocial work environment. These suggest that work characterized by high demands and low control during pregnancy is associated with preterm delivery and low birth weight infants [13-19], although not all studies support this [20]. All together the results do not suffice to asses if special recommendations in relation to the psychosocial work environment are needed for pregnant women. This is also due to relatively small studies (480-800 pregnant women) [13,16,18] and retrospective designs, or use of registers/job titles when assessing psychosocial job strain [13,15,17].

The present project therefore aims to examine if maternal exposure to psychosocial job strain (high demands and low control) measured by questionnaire early in pregnancy is associated with preterm birth, being born small or large for gestational age (SGA/LGA), in a large and prospective Danish birth cohort.

In accordance with Guidelines for Good Epidemiological Practice [21], this protocol outlines the methods that will be used in the study, before the analyses are initiated.

## Methods and design

Population and data material are the Danish National Birth Cohort (DNBC), which has been approved by the Danish National Ethics Board [22]. The DNBC was established in 1996 and includes prospective data from more than 100.000 pregnancies in Denmark. To contribute, the women had to be pregnant, have intention to carry pregnancy to term, reside in Denmark and speak Danish sufficiently well to participate in telephone interviews.

Primary data was collected from 1996-2003. General practitioners invited the women to participate in the cohort study at their first antenatal visit. About 50 percent of all general practitioners in Denmark agreed to take part in the DNBC and about 60 percent of the invited women agreed to participate. The design of DNBC implied four interviews; in week 12-14 and 30-32 of pregnancy and when the child was 6 and 18 months old. The women were, by phone, asked about health, work environment, habits, medication and development and well-being of the child. An English version of all interview guides is available at the DNBC homepage [23]. For further information on the structure and aim of DNBC please refer to Olsen et al., 2001 [22].

According to Danish regulations only studies involving human beings or any kind of human tissue needs approval from the Ethics Committee. As this study is based on data from questionnaires we do not have nor need to have an ethical approval or informed consent [24].

Information on the woman's psychosocial work environment during pregnancy was obtained from the first of the four interviews. Data on pregnancy course and children's health are available partly from interviews and partly from the Danish Medical Birth Register.

### Inclusion criteria

**Figure 1:**
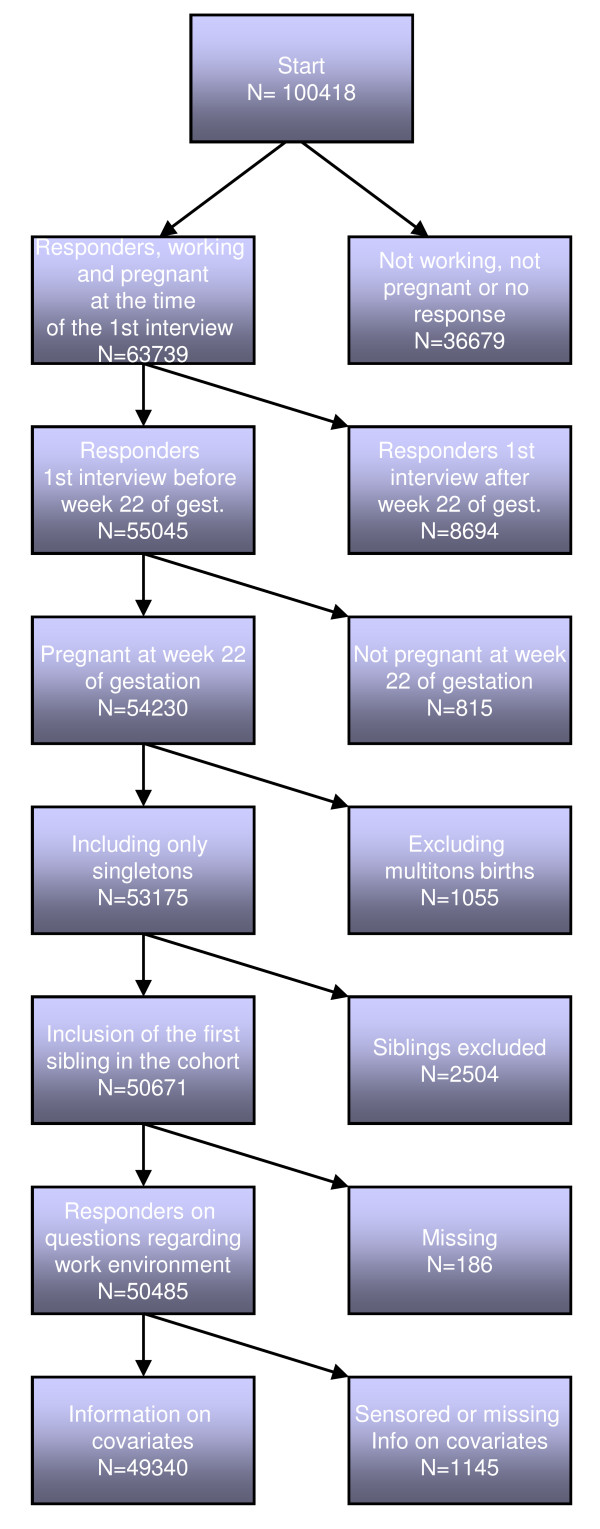
**Flowchart with regard to inclusion/exclusion of pregnancies**.

In total, 100,418 pregnancies were included in the DNBC. In the presented protocol the first inclusion criteria is confirmation in the baseline interview, that the woman is still pregnant and working, which results in 63,739 pregnancies. In order to avoid recall bias, we want the women to answer the question regarding psychosocial work environment before they know the outcome of the pregnancy. This is especially relevant for preterm birth, which is defined as a child delivered after 22 and before 37 completed gestational weeks. Thus 8,694 pregnancies will be excluded because baseline interviews were carried out later than 21 completed weeks of gestation. Subsequently, 815 pregnancies will be excluded because they ended before 22 completed weeks of gestation and therefore per definition (in Denmark) are considered a miscarriage and not of primary interest in this study. Since lowered birth weight and preterm birth may arise from different causes in singletons and multitons, we will only include singleton pregnancies (n = 53,175). To avoid over-representation of gene material by inclusion of siblings, a woman is only allowed to contribute with her first pregnancy in the cohort (n = 50,671). An additional 186 pregnancies will be excluded from the study population due to no response to the question regarding exposure to psychosocial job strain. Finally information on covariates is lacking for 1,145 cases: the final study population will therefore include 49,340 pregnancies. A flowchart of the exclusion process is given in Figure 1.

### Exposure to psychosocial workplace factors

Exposure data concerning psychosocial work environment are collected as self-reported information from the first telephone interview (week 6-21, median 15 weeks), with the response categories: often, sometimes and seldom:

• A172: Do you have too many tasks at your work? (Demand dimension)

• A173: Do you have the opportunity to influence your tasks and working conditions? (Control dimension),

The questions allow assessment of psychosocial load in the working environment according to the dimensions of the Demand Control Model (Job Strain Model [25]).

Based on the answers the women are divided into four job categories related to the dimensions of demand and control: low strain (low demands, high control), active (high demands, high control), passive (low demands, low control) and high strain (high demands, low control). The hypothesis relates to the Job Strain Model by Karasek which has been used previously in DNBC to discuss effects of work-related stress [26,27].

**Figure 2:**
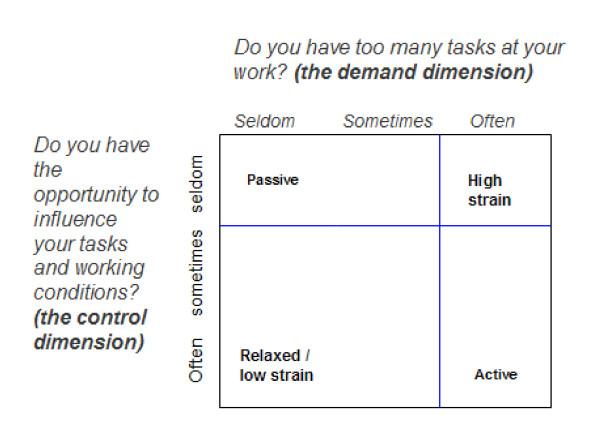
**Division of demands and control related to the questions given in the questionnaire from DNBC**.

To maximise contrast in exposure, the high strain group is defined by those who answered 'often' to high demands and 'seldom' to the question relating to control. The combination of high demands and low control is the primary interest of this study as it predicts mental strain according to the Job Strain Model [28]. The high strain group will be compared with the low strain group which is defined as those who answered 'sometimes' or 'seldom' to high demands and 'often' or 'sometimes' to high control (as in [26,27]). Results from the active and passive group will also be presented. The grouping is illustrated in Figure 2.

For explorative purposes there are furthermore the possibility to study social support in relation to the Job Strain model as introduced by Johnson and Hall in 1988 in the Demand-Control-Support model [29]. This can be done by including question A174 "Do you get any help from colleagues when you have troubles in the work? " with the response categories: often, sometimes and seldom.

### Outcomes

In the present study we aim to examine the potential relationship between psychosocial job strain and perinatal outcomes as SGA, LGA and preterm birth. All the outcomes are defined relative to gestational age. Gestational age (GA) will be calculated as the number of days from "the first day of the last normal menstrual period (LMP)" to the day of birth. LMP is used because the precise time of conception is rarely known [30]. Data come from the Danish Medical Birth Register by use of the personal identification number given to every newborn child in Denmark. The personal idenfication number allows for combining data from the DNBC with other registers in Denmark.

#### Low birth-weight, SGA and LGA

Low birth weight (LBW) is usually defined as a birth weight below 2500 g [30]. Of all liveborn children in Denmark 4.8% had a birth weight below 2500 g in year 2008. Birth weight (BW) has been a much used endpoint in epidemiological studies as it is easy to measure accurately and highly accessible [31]. Furthermore LBW is a powerful predictor of neonatal survival and LBW has been associated with adverse health outcomes later in life, e.g. type 2 diabetes and hypertension [32]. LBW is, however, subject to criticism since it fails to distinguish between preterm and small term babies as birth weight is not only a result of foetal growth, but also of the length of gestation. GA should therefore be taken into account when studying birth weight. This is the case for SGA, as SGA is typically defined as the smallest 10% of babies at each gestational week [30,33]. Likewise, LGA is defined as gestation specific birth weight above the 90^th ^percentile.

In the present study, SGA is defined as the 10% smallest babies at each gestational week in each gender within the present study population. For gestational weeks with less than 10 children in each group (week 22-24), SGA is equal to the lowest birth weight in the group. The same applies to LGA, just the 90^th ^percentile or above is used.

#### Preterm birth

Preterm birth is defined as a delivery occurring before 37 completed gestational weeks. It accounts for approximately 5% of all births in Denmark [34]. Preterm birth is the factor most strongly associated with perinatal and neonatal mortality and morbidity [35]. Babies born preterm are at a higher risk for chronic pulmonary disease [36], cerebral palsy [37] and other neurological disorders [38,39]. The overall proportion of preterm deliveries seems to be increasing in both Denmark [40] and the USA [41].

In the present study, preterm birth will be defined as a delivery after 22 and up to 36 completed weeks of gestation. If analysis indicates statistically significant difference between preterm and term birth in relation to psychosocial strain - the variable will be further divided into three subgroups; extremely preterm (22-27 completed weeks of gestation), very preterm (28-32 completed weeks of gestation), or moderately preterm (33-36 completed weeks of gestation).

Pregnancies that terminate before 22 completed gestational weeks is defined as a miscarriage in Denmark [42], and will therefore not be included in the group of preterm birth, as already described.

### Statistical analysis

The included births will divided into the following categories:

1. Full term and normal weight for gestational age

2. Preterm

3. Full term but small for gestational age (SGA)

4. Full term but large for gestational age (LGA)

With the category 'full term and normal weight for gestational age' as reference (category 1), multinomial logistic regression will be used to estimate odds ratios (OR), with 95% confidence interval (CI), for being in outcome category 1-3 as a function of job-strain (high strain, active and passive versus low strain). The analysis will be conducted with the procedure proc logistic in the computer package SAS version 9.1. A likelihood ratio test will be used to test the overall null-hypothesis, which states that the outcome vector is independent of job-strain. There will be controlled for age, BMI, parity, gestational age at interview, exercise, smoking habits, alcohol habits, coffee consumption, type of work (manual versus non-manual), maternal serious chronic disease as class variables and parents' heights (continuous variable). The categorisation of variables is described below.

Some children will in addition to being preterm also be defined as SGA or LGA. However as only 5% of all cases are preterm and 10% of all cases are SGA, a total of 0.5% of the cases are both preterm and SGA. The expected number of preterm*SGA in the high strain group (the maternal exposure group of primary interest) is therefore less than the number of parameters in the model. Hence, further division of the preterm category is not feasible. An advantage of this outcome categorisation is a clean reference outcome (full term and normal weight for gestational age) with which the three types of cases can be contrasted. In other words the aim is to look at the odds of being preterm versus full term and normal weight for gestational age, the odds of being full term and SGA versus full term and normal weight, and the odds of being full term and LGA versus full term and normal weight. These three separate outcomes will be analyzed together by multinomial logistic regression rather than by separate binomial logistic regressions (one for each outcome). Table 1 shows the planned presentation of results of the analysis.

**Table 1:** Example of a table to be filled out

Exposure/Outcome	Preterm (P = x.xxx)		SGA (P = x.xxx)		LGA (P = x.xxx)	
	**OR**	**95% CI**	**OR**	**95% CI**	**OR**	**95% CI**

High strain						

Passive						

Active						

Relaxed/low strain	1.00	-	1.00	-	1.00	-

#### Significance level

The null-hypothesis will be rejected if P ≤ 0.05. To guard against mass-significance, an estimated odds ratio will only be accepted as statistically significant if i) the overall null-hypothesis has been rejected and ii) the 95% confidence interval of the odds ratio does not include one.

#### Statistical power

**Figure 3:**
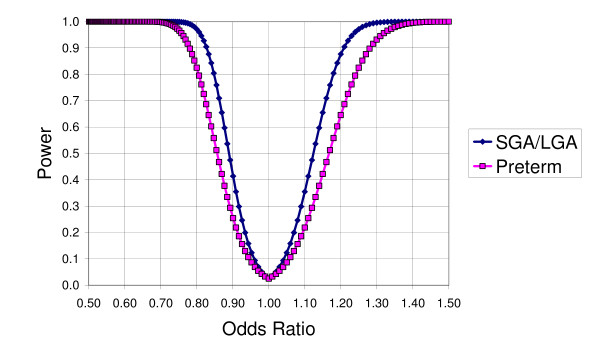
**Power curves for SGA, LGA and preterm birth**.

The presented power calculations focus on the contrast in the high strain versus low strain groups, which is our primary interest. Figure 3 depicts power curves for each of the three response variables SGA, LGA and preterm birth. If for example an odds ratio of 1.3 or higher is considered clinically significant, the analysis will have a 99.6% possibility of identifying a statistically significant association between job-strain and SGA, if the true odds ratio for SGA between these two job-strain categories is higher than 1.3 or lower than 0.77 (or 1.3^-1^). The same holds for the outcome LGA, since the prevalence of LGA, by definition, is the same as it is for SGA, i.e. 10 percent. For preterm birth, the corresponding power is 93.2%.

### Covariates

Below we list, motivate and describe the covariates that will be included in the analysis. In Table 2 the exposure variable is cross-tabulated with each of the covariates. In construction of the covariates coding by other researchers working with the DNBC, frequency tables and meaningful grouping (i.e. recommendations by the WHO) has been taken into account.

**Table 2:** Cross-tabulation between exposure variable and covariates

Characteristics	*High strain*		*Active*		*Passive*		*Low strain*	
	
N = 50485	N	%	N	%	N	%	N	%
	
	3442	6.8	11435	22.7	4427	8.8	31181	61.8
**Maternal age**								
*<25*	318	9.2	656	5.7	494	11.2	1987	6.4
*25-29*	1320	38.4	4108	35.9	1751	39.6	12012	38.5
*30-34*	1341	39.0	4765	41.7	1575	35.6	12325	39.5
*>35*	463	13.5	1906	16.7	607	13.7	4857	15.6
**Parity**								
*0 births*	1603	46.6	5668	49.6	2218	50.1	16193	51.9
*1 birth*	1256	36.5	4150	36.3	1557	35.2	10518	33.7
*≥ 2 births*	580	16.9	1605	14.0	648	14.6	4456	14.3
**Smoking during pregnancy**								
*No*	2346	68.2	8529	74.6	3171	71.6	24331	78.0
*Less than daily*	442	12.8	1314	11.5	501	11.3	3168	10.2
*Daily*	654	19.0	1590	13.9	754	17.0	3669	11.8
Alcohol (units per week*)								
*0*	2009	58.4	6123	53.6	2458	55.5	16506	52.9
*<1*	516	15.0	1787	15.6	742	16.8	5145	16.5
*1-2*	764	22.2	2962	25.9	1067	24.1	8122	26.1
*>2*	147	4.3	546	4.8	155	3.5	1381	4.4
**Coffee**								
*0 cups per day*	1873	54.4	6116	53.5	2527	57.1	17295	55.5
*>0 cups per day*	1568	45.6	53.18	46.5	1998	42.9	13877	44.5
**Physical exercise**								
*None*	2312	67.2	7047	61.6	2934	66.3	18606	59.7
*<3.5 hours per week*	898	26.1	3541	31.0	1227	27.7	10277	33.0
*≥ 3.5 hours per week*	221	6.4	820	7.2	255	5.8	2233	7.2
**BMI**								
*15-18.4*	126	3.7	460	4.0	194	4.4	1224	3.9
*18.5-24.9*	2262	65.7	7943	69.5	2857	64.5	21336	68.4
*25-29.9*	707	20.5	2054	18.0	927	20.9	5902	18.9
*30-50*	294	8.5	786	6.9	378	8.5	2193	7.0
**Type of work**								
*Manual*	2093	60.8	5389	47.1	2045	46.2	11856	38.0
*Non-manual*	1349	39.2	6041	52.8	2378	53.7	19307	61.9
**Maternal diabetes or epilepsy**								
*Neither*	3377	98.1	11238	98.3	4342	98.1	30751	98.6
*either or both*	62	1.8	183	1.6	80	1.8	391	1.3
**Gestational age at interview (weeks)**								
*<16*	1802	52.4	5756	50.3	2363	53.4	16060	51.5
*16 - 21*	1640	47.7	5679	49.7	2064	46.6	15121	48.5

* one unit is equal 12 grams of alcohol which corresponds to one normal beer (33 centilitre), one glass of wine (12,5 centilitre) or 4 centilitre spirits.

#### • Maternal Age

Women older than 35 years have an elevated risk of giving birth to children with low birth weight [43,44]. Information about mother's age at birth is collected from the Danish Medical Birth Register. The variable is calculated by the data-managers from DNBC based on the personal identification number of the mother together with the date of birth of her child. Age is divided into four categories; <25, 25-29, 30-34, >35 years

#### •Parity

Generally women deliver lighter babies in their first pregnancy compared to the following pregnancies [45,46]. As parity could confound the relationship between psychosocial job strain and birth weight (as used in the definition of SGA) it will be included as a covariate in the analyses. Medical parity is the number of times a woman has given birth - thus delivering twins/triplets only counts as one birth as do stillbirths [47]. The parity variable is categorized into: 0, 1, ≥2 births.

#### • Smoking

Several studies show that cigarette smoking is causatively related to low birth weight or SGA; and cigarette smoking is the single most important risk factor for SGA in developed countries [48]. According to the Danish National Board of Health the risk of being born with a birth weight below 2500 g doubles if the mother smoked during pregnancy [49]. In DNBC, several questions were asked related to smoking; A127 "Did you smoke during pregnancy - please also think back to the very beginning of the pregnancy?" with the response-categories "yes", "no", "do not know", and "do not want to answer", and A128 "Do you smoke now?" with the response categories "yes - every day", "yes - less than every day", "no", "do not know", and "do not want to answer". From the answers three categories are constructed; 1) has not smoked during pregnancy, 2) smoked during pregnancy but does not smoke every day (at the time of the interview), 3) smokes every day.

Cross-tabulation of the smoking variable from the first and the second interview during pregnancy shows that 89 percent of the women, who were smoking daily in the first part of the pregnancy also smoked daily during the last part of the pregnancy. The smoking variable is therefore only based on the questionnaire from the early pregnancy where the exposure question also derives from.

#### • Alcohol

It is documented that a large alcohol intake during pregnancy increases the risk of adverse birth outcomes [50,51]. Alcohol consumption will therefore be included as a covariate. The variable is defined as the number of drinks/units per week, which is the total sum of beers, glasses of wine and glasses of spirits consumed per week, based on the answers to the questions; A138 "How many normal beers do you drink per week?", A140 "How many glasses of wine do you drink per week?", and A143 "How many glasses of spirits do you drink per week?". If a person answered less than 1 glass of alcohol per week, this is defined as equal to 0.5 glasses per week. The categorization of the alcohol-variable is; 0 units of alcohol per week, less than 1 unit of alcohol per week, 1-2 units of alcohol per week, and more than 2 units of alcohol per week.

#### • Coffee

A high caffeine intake (>300 mg per day) during pregnancy has been associated with a reduction in birth weight of 100-200 g compared to that in women with a low caffeine intake [52] although not all studies support this [53,54]. DNBC data provides information on daily cups of coffee with the question: A136 "How many cups of coffee do you drink per day?"

In the DNBC questionnaire, the questions immediately prior to coffee consumption had answer categories relating to consumption per week. In contrast the categories for coffee consumption were to be given per day. Such shifts between answer categories may create faulty answers, i.e. the women answered per week, but answers were categorised per day. In the present survey, it was obvious that some of the responders meant coffee consumption per week instead of per day as the number of cups ranged between 0 and 69. Due to miscategorization, it was decided to construct a dichotomic yes/no variable.

#### • Physical exercise

Health authorities in several countries recommend physical exercise during pregnancy, due to its beneficial effects on adverse health outcomes as pre-eclampsia [55] and gestational diabetes [56]. Results on potential beneficial effects for the foetus are diverging - some studies report increased risk of miscarriage when exercising early in pregnancy [57] where others do not observe indications on adverse effects of exercise related to preterm birth [58].

The applied variable is constructed on the basis of the work of Juhl et al [58]. Here several of the questions in the DNBC questionnaire were used to construct a variable, which estimates how many minutes per week a woman was engaged in exercise: A148 "Do you get any kind of exercise during pregnancy?", A149 "What kind of exercise?" and A151 "How many minutes at a time do you do ____ (answer from A149)?". Questions were repeated if the woman participated in several types of exercise. Hence three categories are constructed; 0 minutes, less than 31/2 hours and 31/2 hours or more exercise per week. The cut-point of 31/2 hours is selected based on recommendations from the health authorities [58].

#### • Maternal BMI (pre-pregnancy)

An increasing number of women are overweight when they become pregnant. Numbers from the Danish National Board of Health show that more than one third of the pregnant women in 2008 had a BMI above 25 [59]. Maternal overweight and obesity are known risk factors for congenital malformations in the offspring [60] and elective preterm deliveries [61]. In accordance with the WHO, BMI will be classified as; underweight (15 ≤ BMI < 18.5), normal (18.5 ≤ BMI < 25), overweight (25 ≤ BMI < 30) and obese (30 ≤ BMI < 50). Women with a pre-pregnancy BMI under 15 and over 50 will be excluded in this study as such extreme BMIs must be proposed to represent such an adverse environment for the foetus that possible effects on the child may be due to this rather than an adverse psychosocial work environment.

#### • Type of work

Women with manual work might have a different risk of adverse pregnancy outcomes than those performing non-manual work. In addition this factor is associated with a person's opportunity to influence work tasks and working conditions [62]. The variable "Type of work" is therefore constructed from question A175 "Is your work physically strenuous?" with the response categories: "often", "sometimes", "seldom", "do not know", "irrelevant" and "do not want to answer". Type of work is categorized as manual if the answer is "often" or "sometimes" and non-manual if the answer is "seldom".

#### • Maternal diabetes/epilepsy

Some diseases may affect pregnancy or foetal development either as a result of the disease itself or due to pharmacological treatment. Diabetic women display increased risk of congenital malformations, obstetric complications and neonatal morbidity [63-65]) regardless of type of diabetes [65]. In relation to epilepsy most diagnosed women need antiepileptic drug therapy during pregnancy to diminish the risk of seizures which can affect both the mother and the unborn child negatively [66]. Antiepileptics are also known to increase the risk for congenital malformations [67]. A variable called diabetes/epilepsy is therefore constructed based on question A088 "Have you ever had any serious disease that we have still not talked about, for instance, heart disease, epilepsy or diabetes?" The answer variable is a text string, from which women afflicted by the diseases are identified. The two diseases are combined into a single variable due to very few cases in the study-population and since the effect of each of the diseases on the birth outcomes is not of primary interest in the study. No distinction between pregestational and gestational diabetes will be made.

#### • Gestational age at interview

One of the inclusion requirements was that the women should be working at the time of the interview. Such requirements are associated with a healthy worker effect. To require that a women should be working in e.g. the tenth week of the pregnancy is however not the same as requiring that a women should be working in the 19^th ^week. To crudely control for potential differences due to the healthy worker effect, a variable 'gestational age at interview' is constructed, with categories <16 weeks and 16 - 21 weeks.

#### • Parents' heights

We assume that the probability that a child is LGA or SGA at birth depends on how tall the parents are. For control of this parameter a variable will be constructed as follows:

First the heights of each parent are normalized by the equations: , 

where *x_m _*is the height of the mother,  is the mean height of all of the interviewed mothers (N = 82,836) and *σ_m _*is the standard deviation. The variables  and *σ_f _*refer to the corresponding values among the fathers (N = 74,066).

Then a combined variable is formed by averaging the two z-values. This combined variable will be used in the analysis. The father's height is missing in approximately 10% of the interviews. In these cases the z-value of the mother is used as a proxy for the combined z-value. The mean height among the mothers was 168.7 cm with a standard deviation of 6.09 cm. For the fathers these values are 181.9 and 6.98 cm.

## Discussion

We have chosen to present the protocol before undertaking the actual analyses. This is done to improve the quality and integrity of the study by including reviewers' comments prior to analysis in order to ensure that design is not to be changed once data analysis has been performed.

The aim of the presented protocol is to examine the relationship between psychosocial job strain and preterm birth, SGA and LGA. The analyses will be made from a public health perspective. We want to know if women with a work characterised by high demands and low control are at increased risk for the above mentioned pregnancy outcomes, compared to women whose work is characterised by low demands and high control. All kinds of interventions take place throughout the pregnancy, planned as well as incidental. At work, initiatives may be taken, both by colleagues and management, to relieve some of the strain associated with the work of the pregnant woman. Other interventions might originate from the woman herself (e.g. absence from work) or by people in the health care system as a consequence of the repeated health examinations that are offered during the pregnancy in Denmark. From a public health perspective the primary interest is not if job strain has an effect per se but if an effect of job strain resides after preventive measures have been taken, i.e. does the preventive system work, and are the measures taken sufficient to protect the woman. Furthermore we have chosen to initiate the statistical analysis with a single overall multinomial logistic regression. Although the investigated endpoints do probably arise for different underlying mechanisms such an approach is associated with several advantages. The most important advantage is increased power [68]. The multinomial regression has a chance of detecting a significant relationship between the exposure and outcome categories even if none of the estimated odds ratios is significant in itself. Furthermore, multinomial logistic regression decreases the probability of false positive findings (none of the findings will be deemed statistically significant unless the overall null-hypothesis is rejected).

The prospective nature of the design and the high number of participants strengthen the study. The large power allows for interpretable results regardless of whether or not the hypotheses are confirmed. By handling all outcomes simultaneously in a single model, mass-significance problems are eliminated. The hypotheses are theoretically grounded for preterm birth and SGA. Reviews indicate a relationship between severe maternal stress and reduced birth weight [69], and association between maternal and foetal stress, activation of cells in the placenta and production of corticotrophin-releasing hormone resulting in preterm deliveries [70]. Status of the LGA hypothesis is of a more exploratory nature. Gestational age is part of the definitions of preterm birth, SGA and LGA. Gestational age is calculated from the date of the last menstrual period which is not a very precise measure. The effect of such measurement errors is, however, diminished since we are dealing with ratios rather than prevalences. We have no reasons to believe that misclassification depends on exposure to job strain.

It can be discussed if some of the included covariates might be mediating factors in a causal path between work-environment and risk for adverse birth outcomes. Psychosocially strenuous work will, for example, make it more difficult for a person to stop smoking [71]. It has also been shown that stress is probably associated with alcohol use [72]. While omitting such variables would render the model under-adjusted, an inclusion might over-adjust. From a conservative viewpoint, it is, however, better to over-adjust than to under-adjust. Another point for discussion is the potential selection bias associated with volunteer participation to the cohort and with the requirement that the women should speak Danish well enough to participate in telephone interviews. Due to this there might be certain groups of women on the Danish Labour Market who are not represented in this study. Also the women were not followed continuously throughout the pregnancy. Exercise habits at the time of the interview might, for example, not be representative for exercise habits at other periods of pregnancy.

In summary, the validity of the results and conclusions of the project will be strengthened by this protocol. Since the hypotheses, statistical model and significance level are defined and peer-reviewed before we look at any results, the analysis will be free from hindsight bias.

## Abbreviations

BMI: Body Mass Index; CI: Confidence Interval; DNBC: Danish National Birth Cohort; GA: Gestational Age; HPA-axis: Hypothalamic-Pituitary-Adrenal axis; LGA: Large for Gestational Age; LBW: Low Birth Weight; LMP: The first day of the Last normal Menstrual Period; OR: Odds Ratio; SGA: Small for Gestational Age

## Competing interests

The authors declare that they have no competing interests.

## Authors' contributions

ADL, KSH and HHA designed and wrote the first draft. This has been modified and adapted by AMT, CO, JPB. All authors read and approved the final manuscript.

## Pre-publication history

The pre-publication history for this paper can be accessed here:

http://www.biomedcentral.com/1471-2458/11/255/prepub
